# Modelling success after perinatal post-haemorrhagic hydrocephalus: a single-centre study

**DOI:** 10.1007/s00381-022-05597-2

**Published:** 2022-07-07

**Authors:** Saeed Kayhanian, Jonathan Perry Funnell, Katharina Zühlsdorff, Ibrahim Jalloh

**Affiliations:** 1 Department of Neurosurgery, Addenbrooke’s Hospital, Cambridge, CB2 0QQ UK; 2 Department of Psychology, University of Cambridge, Cambridge, UK; 3 Department of Clinical Neurosciences, University of Cambridge, Cambridge, UK

**Keywords:** Post-haemorrhagic hydrocephalus, Shunt, Perinatal hydrocephalus, Generalised linear model

## Abstract

**Introduction:**

Post-haemorrhagic hydrocephalus is common amongst premature infants and one of the leading indications for paediatric cerebrospinal fluid (CSF) diversion. Permanent CSF diversion is often delayed until the infant is older but there is no clear consensus on the timing for this. The outcomes for permanent shunting in this patient group are poor, with higher rates of failure and infection compared to other aetiologies of hydrocephalus.

**Methods:**

We conduct a single-centre retrospective review of infants with post-haemorrhagic hydrocephalus requiring a permanent shunt insertion over a 5-year period. Demographic and clinical data from time of shunt insertion were collected and used to generate generalised linear models (GLMs) to predict shunt success at 12 months after insertion.

**Results:**

Twenty-six infants underwent permanent shunting in this period for post-haemorrhagic hydrocephalus, with 10 suffering shunt failure within the first 12 months. The best-performing GLM was able to predict shunt success with a sensitivity of 1 and specificity of 0.90, with head circumference, weight, and corrected age at the time of shunt insertion being the most significantly associated variables for shunt success in this model.

**Conclusion:**

Our proof-of-principle study suggests that highly accurate prediction of shunt success for infants with post-haemorrhagic hydrocephalus is possible using routinely available clinical variables. Further work is required to test this model in larger cohorts and validate whether pre-operative use can improve outcomes for this patient group.

## Introduction

Post-haemorrhagic hydrocephalus (PHH) is common amongst premature infants and the leading cause of acquired paediatric hydrocephalus in Europe and North America [1, 2]. PHH develops after intraventricular haemorrhage (IVH), caused by bleeding from the fragile germinal matrix in premature infants. IVH occurs in around a quarter of premature infants and an estimated 15% of these infants suffering IVH develop hydrocephalus that requires permanent CSF diversion [3, 4]. This permanent CSF diversion is often delayed until the infant is older, with temporising measures undertaken to reduce CSF volume in the interim, including the insertion of ventricular access devices and ventriculo-subgaleal shunts.

Despite its prevalence, there remains considerable uncertainty about the optimal management of PHH, both with regard to the timing of permanent shunt insertion and the method of managing CSF volume before any permanent shunting [5, 6]. This clinical uncertainty is borne in the context of poor outcomes for this cohort, with higher rates of shunt failure and neurodevelopmental disability compared to patients with other aetiologies of paediatric hydrocephalus [7–9].

In this retrospective cohort study, we set out to identify clinical characteristics that are associated with shunt success in patients with PHH.

## Methods

We conducted a single-centre, retrospective cohort study of consecutive infants with PHH. We searched operative records for primary shunt insertion at Addenbrooke’s Hospital, over the 5-year period between January 2015 and December 2019. We identified patients undergoing primary shunt insertion for PHH and extracted data from the electronic health record system. Demographic and surgical data were collected, as well as clinical characteristics including gestational age at birth, Papile Grade of Intraventricular Haemorrhage, previous temporising measures employed, corrected age, weight, and head circumference (HC) at the time of shunt insertion. The date of shunt failure, if applicable, was recorded. The outcome at 12 months was dichotimised as success or failure for the original shunt.

Generalised linear models (GLMs) were applied to the data to identify parameters that predict this dichotimised outcome at 12 months. Four models using different combinations of clinical characteristics were tested, all of which included gender, corrected age, and Papile Grade. Three of the four models also included weight and head circumference, one model further included gestational age, and the final model further included the method of previous temporising measures. Model fit was compared using the Akaike information criterion (AIC), Bayesian information criterion (BIC), and *p*-values. Lower AIC, BIC, and *p*-values indicate a better model fit. The contribution and directionality of individual parameters within each model were evaluated using *t*-statistic values. All statistical analyses were performed using R: A Language and Environment for Statistical Computing [12].

## Results

### Demographics

Twenty-six patients underwent primary shunt insertion for PHH in this period. The decision to progress to shunting in this period was made by treating clinician based on persistently enlarged ventricular index (> 97th centile + 4 mm) and/or a regular, on-going need for CSF withdrawal to control head circumference or the signs of raised intracranial pressure. The first two CSF withdrawals were by lumbar puncture and further requirement led to insertion of a ventricular access device.

All patients received ventriculo-peritoneal shunts with NSC Strata valves (Medtronic, USA). Valves were implanted in an in-line configuration and secured to the periosteum using a nylon stitch. Clinical data of interest was available for all patients.

Ten patients suffered from shunt failure within the first 12 months. The most common cause of failure was migration of the proximal catheter (*n* = 4). Other causes of failure included infection (*n* = 2), obstruction (*n* = 2), migration of the distal catheter (*n* = 1), and proximal wound breakdown (*n* = 1). The mean time to shunt breakdown was 5.1 ± 3.5 months. The cohort’s demographic and clinical data is summarised in Table 1.

**Table 1:** Demographics of patients at time of permanent shunt insertion

	Successful (*n* = 16)	Failed (*n* = 10)	*p*-value
Gestational age at birth (weeks)	29.5 ± 4.9	28.3 ± 4.1	0.75
Male [%]	10 [63]	6 [60]	0.90
Corrected age (weeks)	7.3 ± 9.1	3.9 ± 10.1	0.21
Weight (kg)	4.9 ± 1.6	4.3 ± 1.8	0.36
Head circumference (cm)	40.1 ± 4.3	41.1 ± 6.1	0.59
Papile Grades (range) [mode]	II–IV [III]	III–IV [III]	N/A
Previous temporising measures (*n*):			N/A
Ventricular access device	13	9	
Subgaleal shunt	1	1	
Lumbar puncture	1	0	
None	1	0	

Mean values ± SD are given for age, weight, head circumference

*p*-values show significance testing by Mann–Whitney *U* test

The majority of patients in this cohort received temporising measures to manage hydrocephalus by serial CSF removal via an inserted ventricular access device (*n* = 22), further summarised in Table 1.

### Generalised linear models

**Fig. 1:**
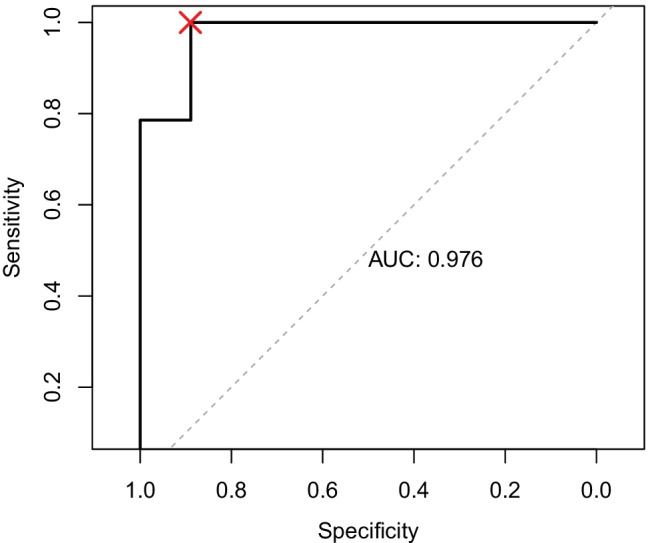
Receiver operating characteristic curve showing the performance of model 2, with a sensitivity = 1.0 and specificity = 0.9 at the point indicated

A summary of the performance of the four GLMs is presented in Table 2. Model 2 (which accounted for Papile Grade, corrected age, sex, weight, and head circumference) provided the best fit to the data, with the lowest scores across all three statistical measures of model fit: AIC, BIC, and *p*-values. The area under the receiver operating characteristic curve (AUROC) for predicting shunt success using model 2 was 0.976, with the optimal sensitivity = 1 and specificity = 0.90 (Fig. 1). Within model 2, head circumference was found to have the most significant effect (*t*(22) =  − 2.55, *p* = 0.021), with shunt success at 12 months associated with a smaller head circumference. Corrected age and weight were also found to significantly affect this outcome (*t*(22) = 2.34, *p* = 0.033 and *t*(22) = 2.05, *p* = 0.039, respectively), with patients who were older and a greater weight at the time of shunt insertion more likely to have successful permanent shunts at 12 months.

**Table 2:** Summary of the four generalised linear models. Four models with different predictive variables were compared

Model number	Predictive variables	AIC	BIC	*p*-value
1	PG + CA + Sex	38.36	45.18	0.12
2	PG + CA + Sex + Weight + HC	24.85	33.94	0.00052
3	PG + CA + GA + Sex + Weight + HC	26.58	36.80	0.00093
4	PG + CA + Sex + Weight + HC + TM	27.89	39.24	0.0013

*AIC* The Akaike information criterion, *BIC* Bayesian information criterion, and *p*-values provide a measure of model fit, with lower values indicating better model fit

*PG* Papile Grade, *CA* corrected age, *HC* head circumference, *GA* gestational age, *TM* temporising measures

## Discussion

The outcomes for permanent shunting after perinatal PHH are poor and, as indicated in Table 1, there is no single clinical variable that is significantly predictive of shunt success or failure. However, we show that generalised linear models using simple, clinically relevant parameters concurrently are able to predict shunt success to a high degree of accuracy. The best-performing GLM points towards head circumference, corrected age, and weight at the time of shunt insertion as the most significant variables, when taken as part of a model that accounts for a total of five parameters concurrently.

This is a proof-of-principle study but the results suggest that, by using larger datasets from multiple centres, there is scope to use similar modelling to derive precise thresholds of simple bedside parameters, such as head circumference, age, and weight, to optimise the timing for permanent shunt insertion and so maximise the chances of success.

Increasing age and weight were both positively associated with shunt success but increasing head circumference was negatively associated with this outcome. The negative association with head circumference is likely to be related to the severity of hydrocephalus that has developed in the infant by the time of shunt insertion. Taken together, this modelling suggests that while it is advisable to defer permanent shunting until the infant is older, as has been suggested previously, this must be balanced with the degree of hydrocephalus that can develop in this period and patients should be very carefully temporised in the interim to avoid excess increases in head circumference [9, 10].

This was a single-centre, retrospective study and is limited by the small size of the dataset. In particular, the majority of patients were temporised using the same method of serial CSF withdrawal from an inserted ventricular access device — the standard practice in our centre during this period. This study is unable therefore to draw any conclusions about the best method of temporising measure, which remains an important outstanding question in this cohort [11]. This study also did not control for surgical factors, such as operating surgeon and time of day for operation, due to limited numbers. However, the single-centre design was advantageous in controlling for operating procedures, which are standard in our centre across cases, for instance for the type of shunt inserted and the intra-operative antibiotics received by all patients. Larger cohort studies are warranted to validate these findings and develop this modelling further.

## Conclusion

There is considerable scope to improve outcomes for infants who develop post-haemorrhagic hydrocephalus. We demonstrate that smaller head circumference, as well as increased weight and corrected age, at the time of shunt insertion is significantly associated with shunt success in this cohort using a generalised linear model. Larger cohort studies will be required to validate this finding but our proof-of-principle work suggests there is potential to develop an accurate shunt success model to improve outcomes for this patient population.

## Data Availability

All data generated or analysed during this study are included in this article. Further enquiries can be directed to the corresponding author.
